# Cigarette smoking during pregnancy and adverse perinatal outcomes: a cross-sectional study over 10 years

**DOI:** 10.1186/s12889-022-14881-4

**Published:** 2022-12-21

**Authors:** Baptiste Tarasi, Jacques Cornuz, Carole Clair, David Baud

**Affiliations:** 1 Materno-Fetal and Obstetric Research Unit, Woman-Mother-Child Department, University Hospital of Lausanne, CHUV, 1011 Lausanne, Switzerland; 2 Department of Ambulatory Care, Center for Primary Care and Public Health (Unisanté), University of Lausanne, 1011 Lausanne, Switzerland; 3 Department of Training, Research and Innovation, Center for Primary Care and Public Health (Unisanté), University of Lausanne, 1011 Lausanne, Switzerland

**Keywords:** Smoking during pregnancy, Perinatal outcomes, Preterm birth, Birthweight, Intrauterine growth restriction

## Abstract

**Background:**

It has been shown that active exposure to tobacco is associated with adverse pregnancy outcomes including, but not limited to, intrauterine fetal death, reduced fetal weight, and higher risk of preterm birth. We want to investigate these effects in a high-income country.

**Methods:**

This cross-sectional study examined 20,843 pregnant women who delivered over 10 years at the Maternity Hospital of the Centre Hospitalier Universitaire Vaudois (CHUV) in Lausanne, Switzerland. The objective was to evaluate a dose–response relationship between daily cigarette use during pregnancy and possible adverse perinatal outcomes. The social and clinical characteristics as well as obstetric and neonatal outcomes were compared between the smoking and the non-smoking groups. Adjusted odds ratios (aOR) and trend analyses (p_trend_) were calculated.

**Results:**

Nineteen thousand five hundred fifty-four pregnant women met the inclusion criteria and 2,714 (13.9%) of them were smokers. Even after adjusting for confounding factors, smoking during pregnancy was associated with preterm birth, birthweight < 2500 g, intrauterine growth restriction, neonatal respiratory and gastrointestinal diseases, transfer to the neonatal intensive care unit, and neonatal intensive care unit admissions > 7 days. Intrauterine death and neonatal infection were associated with heavy smoking (≥ 20 cigarettes/day). Smoking appeared to be a protective factor for pre-eclampsia and umbilical cord arterial pH below 7.1. A significant trend (p_trend_ < 0.05) was identified for preterm birth, intrauterine growth restriction, birthweight < 2500 g, umbilical cord arterial pH below 7.1, transfers to our neonatal intensive care unit, and neonatal intensive care unit admissions more than 7 days.

**Conclusion:**

Cigarette smoking is associated with several adverse perinatal outcomes of pregnancy with a dose-dependent effect.

## Background

Among adults, the consequences of cigarette use are well known and can lead to cardiovascular, pulmonary, and oncological diseases as well as other chronic illnesses [1]. These negative health consequences are remote in time and therefore do not always cause sufficient immediate concern to motivate smoking cessation, especially in younger individuals [2]. The number of smokers worldwide in 2019 was estimated to be 1.14 billion, corresponding to 7.69 million deaths and 200 million DALYs (Disability Adjusted Life Years). Globally, the proportion of smokers is much lower among women with 6.62% of female individuals identified as smokers compared to 32.7% of male individuals. However, this proportion is considerably higher among women in high-income countries with 17.6% of women compared to 26.9% of men identifying as smokers [3].

There is evidence that women are more likely to discontinue cigarette use during their pregnancy [4]. The global prevalence of smoking during pregnancy is estimated to be 1.7% [5]. This proportion, also evaluated in 2018, is significantly higher in high-income countries, reaching 7.2% in the USA [6] and 8.1% in Europe [5]. These numbers should be interpreted cautiously as up to 25% of pregnant women with cigarette use prior to pregnancy incorrectly indicated that they ceased smoking during pregnancy [7]. Pregnant women with a lower level of education and those who experience an unplanned pregnancy have a higher prevalence of smoking and a lower probability of quitting [8, 9].

The effects of smoking during pregnancy have been the subject of numerous studies and have been associated with many adverse perinatal outcomes. Specifically active exposure to tobacco has been shown to be associated with a dose–response relationship to adverse outcomes such as preterm birth (birth before 37 weeks of pregnancy) [10–12], reduced birth weight [13, 14], with the reduction in fetal measurements occurring after the first trimester [15], and transfer to a neonatal intensive care unit [16]. Smoking has also been associated in a dose-dependent manner with an increased risk of intrauterine fetal death [17–20]. In contrast to adverse outcomes cited, smoking has been identified to be a protective factor against pre-eclampsia [21, 22]. Regarding the neonatal impact, smoking during pregnancy can alter fetal lung development and lead to respiratory problems [23, 24]. Long term, fetal exposure to smoking during pregnancy can result in more frequent development of gastrointestinal pathologies [25].

In summary, many studies have already investigated adverse obstetric and neonatal outcomes [26, 27]. However, not all of them included a large sample from a single center or adjusted their results to account for potential confounding factors. In addition, many studies have focused only on a single adverse outcome. For example, Soneji et al. focused their study on prematurity [12], and Larsen et al. focused mainly on birth weight [13]. If we take the main studies found in the literature that focused on several outcomes, Ratnasiri et al. did not focus on neonatal outcomes and did not evaluate a potential dose–response [28]. Finally, the well conducted research of Li et al. did not focus on several key outcomes including the risk of pre-eclampsia or neonatal infections, pulmonary pathologies, or gastrointestinal pathologies and did not evaluate a potential dose–response as well [29].

For all these reasons, we firstly aimed to assess multiple obstetric and neonatal outcomes associated with cigarette smoking during pregnancy within a single and large Swiss obstetric cohort with prospectively collected data. Some have already been studied, others not. Secondly, we want to evaluate a potential dose–response relationship between the quantity of cigarette use and adverse outcomes.

## Methods

This cross-sectional study utilized our obstetrical database at the Maternity Hospital of the Centre Hospitalier Universitaire Vaudois (CHUV) in Lausanne, Switzerland, where 20,843 pregnant women gave birth between 1997 and 2006. Data available in this database include demographic, labor, and delivery information, as well as maternal and neonatal outcomes.

All information regarding patient health and pregnancy was collected at the time of admission to the hospital, with the majority occurring at the time of admission for delivery or, for some, at the time of admission to the antepartum unit in the case of complicated pregnancies. A medical history was taken for each patient presenting to the hospital by the obstetrical care provider. If urgent care was required, the history was postponed to an appropriate time during the hospitalization. Our computer system did not permit closure of a patient file that did not include all the mandatory information, including smoking habits. This information was collected verbally with the following question: "Do you smoke cigarettes daily?" with a dichotomous “yes/no” answer. If the answer was “yes”, the number corresponding to the current consumption was then requested by the computer system. The number of cigarettes consumed thus represents usage in the late third trimester, and does not take into account variation of smoking during pregnancy.

Regarding neonatal data, all information was added to our database at the end of the stay by the neonatologists and/or the obstetricians. All women whose records contained all the data needed for our study were included regardless of mode of delivery. The exclusion criteria were as follows: women under 18 years of age or women with multiple pregnancies. The quality of this database of prospectively collected data has already been described elsewhere (cross-check congruent data in 98.2–99.8% of cases) [30].

The following social and clinical characteristics were extracted from the database: daily cigarette use, maternal age, country of birth, marital status, parity, previous pregnancy loss, education, professional status, health insurance, and the presence of significant psychosocial difficulties. The latter was defined as pregnant women referred for a dedicated indication for consultation associated with challenging psychosocial circumstances (psychiatric pathologies, alcohol or drug abuse, etc.…). We assessed the following obstetric and neonatal outcomes: delivery mode, pre-eclampsia, intrauterine death, neonatal death, preterm birth, intrauterine growth restriction, birthweight, umbilical cord arterial pH, APGAR score at 5 min, neonatal infection, hypoglycemia, cerebral hemorrhage or convulsion, jaundice, neonatal anemia, respiratory diseases (including pulmonary infection, pneumothorax, apnea, and hyaline membrane disease), gastrointestinal diseases (including feeding difficulties, occlusive syndrome, digestive hemorrhage, necrotizing enterocolitis, diarrhea, and vomiting), transfers to our neonatal intensive care unit, and neonatal intensive care unit admissions longer than 7 days.

The social and clinical characteristics, as well as the obstetric and neonatal outcomes, were compared between the smoking and non-smoking pregnant women. For the same comparisons, the group of smoking pregnant women was also divided into 3 subgroups according to their daily cigarette usage (< 10/day, ≥ 10/day, and ≥ 20/day). The p-value for each clinical and social characteristic, comparing smokers and non-smokers, was calculated using a Chi-squared test. Logistic regression models to assess the association between smoking and obstetric and neonatal outcomes were built and odds ratios were calculated (aOR), adjusted for maternal age, country of birth, marital status, parity, previous pregnancy loss, education, professional status, psychosocial difficulties and insurance. For some outcomes, such as birth weight, intrauterine growth restriction, umbilical cord arterial pH, APGAR score at 5 min, respiratory diseases, gastrointestinal diseases, neonatal infection, hypoglycemia, cerebral hemorrhage or convulsion, jaundice, neonatal anemia, and transfers to or stay in our neonatal intensive care unit, the odds ratios were also adjusted for the gestational age as these outcomes can occur more frequently in preterm neonates. For the calculation of adjusted estimators in multivariate logistic regression models, the baseline variables that significantly differed between both the groups (confounders) or those that are known risk factors for adverse outcomes were included in the models.

Finally, trend analyses (p_trend_) were also calculated, using the Cochran-Armittage test, for all the outcomes examined to evaluate a potential dose–response relationship according to the number of daily cigarettes consumed.

Statistical analyses were performed using STATA 16 (Stata Corporation, College Station, USA).

The study was carried out in accordance with relevant guidelines and regulations (Declaration of Helsinki). This study was approved by the local IRB (Ethical Commission of the Canton of Vaud, Switzerland, protocol no. 101/08).

## Results

**Fig. 1:**
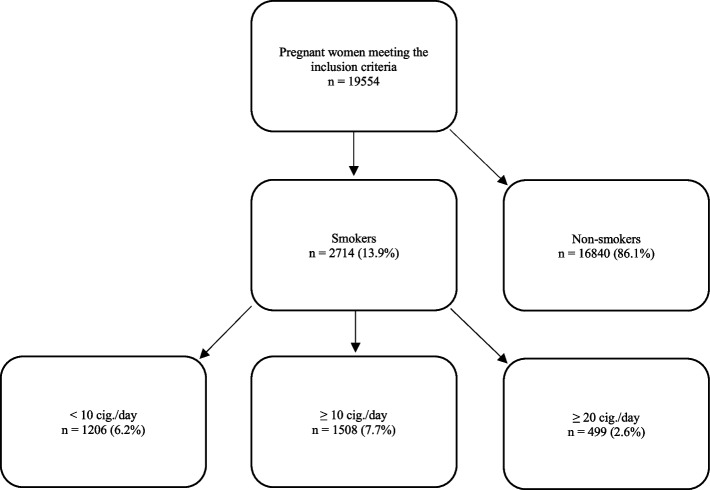
Classification of pregnant women according to the number of cigarettes consumed per day

Over a period of 10 years, 19.554 pregnant women met the inclusion criteria. Among them, 16,840 (86.1%) identified as non-smokers and 2,714 (13.9%) identified as smokers (Fig. 1).

The prevalence of pregnant women who reported cigarette use was higher among pregnant women of Swiss origin, single, divorced, or widowed, those who have had a previous spontaneous abortion, those with significant psychosocial difficulties, and nulliparous pregnant women (Table 1).

**Table 1:** Participant characteristics

	**Total**	**Smokers**		**Non-Smokers**		***p*-value** **(comparing smokers and non-smokers)**
	**(n)**	**(n)**	**(%)**	**(n)**	**(%)**	
Total	19,554	2714	13.9	16,840	86.1	
**Maternal age**						
< 25	4040	732	18.1	3308	81.9	< 0.001
26—30	5889	826	14	5063	86	
31—35	6156	739	12	5417	88	
> 35	3469	417	12	3052	88	
**Origin**						
Swiss	7307	1228	16.8	6079	83.2	< 0.001
Europe	7479	1157	15.5	6322	84.5	
Other	4768	329	6.9	4439	93.1	
**Marital status**						
Married	16,525	1975	12.0	14,550	88	< 0.001
Single/divorced/widowed	3029	739	24.4	2290	75.6	
**Parity**						
Multiparous	10,409	1387	13.3	9022	86.7	0.017
Nulliparous	9145	1327	14.5	7818	85.5	
**Pregnancy loss**						
No abortion	13,434	1687	12.6	11,747	87.4	< 0.001
Previous abortion	6120	1027	16.8	5093	83.2	
**Education**						
Non-academic studies	18,016	2630	14.6	15,386	85.4	< 0.001
Academic studies	1538	84	5.5	1454	94.5	
**Professional Status**						
Employed	16,113	2194	13.6	13,919	86.4	0.021
Unemployed	3441	520	15.1	2921	84.9	
**Health Insurance**						
Minimal insurance	18,804	2664	14.2	16,140	85.8	< 0.001
Private insurance	750	50	6.7	700	93.3	
**Psychosocial difficulties**						
No	19,158	2621	13.7	16,537	86.3	< 0.001
Yes	396	93	23.5	303	76.5	

After adjustment for confounding factors, smoking during pregnancy was associated with preterm birth (aOR 1.16 [95%CI 1.03–1.31]), birthweight < 2500 g (aOR 1.78 [95%CI 1.53–2.08]), small for gestational age (aOR 1.83 [95%CI 1.64–2.05]), respiratory diseases (aOR 1.32 [95%CI 1.13–1.56]), gastrointestinal diseases (aOR 1.63 [95%CI 1.11–2.42]), transfers to the neonatal intensive care unit (aOR 1.44 [95%CI 1.26–1.63]), and neonatal intensive care unit admission > 7 days (aOR 1.64 [95%CI 1.42–1.90]). These associations were stronger in the groups of women with higher number of cigarettes consumed per day. Intrauterine death (aOR 1.98 [95%CI 1.01–3.89]) and neonatal infection (aOR 1.53 [95%CI 1.05–2.22]) were only associated with heavy smoking (≥ 20 cigarettes/day) but not with lower smoking exposure. In contrast, smoking appeared to be a protective factor for pre-eclampsia (aOR 0.62 [95%CI 0.44–0.88]) and umbilical cord arterial pH below 7.1 (aOR 0.65 [95%CI 0.50–0.86]). Rate of cesarean section, neonatal deaths and other neonatal outcomes such as an APGAR score below 7 at 5 min, hypoglycemia, cerebral hemorrhage or convulsion, jaundice, and neonatal anemia showed no significant differences between the smoking and the non-smoking groups (Table 2).

**Table 2:** Association between obstetric and neonatal outcomes and smoking status in pregnant women as well as dose-dependent relationship with quantity of cigarettes

	**Pre-eclampsia**		**Cesarean section**		**Preterm birth**		**IUGR**		**Birthweight < 2500 g**		**Intrauterine death**	
**Cig./day**	**Prevalence %**	aOR (95% CI) ^a^	**Prevalence %**	aOR (95% CI) ^a^	**Prevalence %**	aOR (95% CI) ^a^	**Prevalence %**	aOR (95% CI) ^b^	**Prevalence %**	aOR (95% CI) ^b^	**Prevalence %**	aOR (95% CI) ^a^
Non-smokers	2.1	1	25.6	1	12.0	1	11.2	1	11.0	1	0.92	1
Smokers	1.3	0.62 (0.44–0.88)	27.2	1.08 (0.99–1.19)	14.2	1.16 (1.03–1.31)	19.2	1.83 (1.64–2.05)	16.7	1.78 (1.53–2.08)	0.88	0.87 (0.56–1.36)
< 10 cig./day	1.1	0.51 (0.29–0.90)	26.5	1.15 (1.00–1.31)	11.2	0.92 (0.77–1.12)	16.9	1.57 (1.34–1.84)	13.6	1.61 (1.28–2.01)	0.41	0.43 (0.17–1.05)
≥ 10 cig./day	1.5	0.71 (0.46–1.09)	27.7	1.15 (0.99–1.33)	16.6	1.35 (1.17–1.57)	21.0	2.06 (1.79–2.36)	19.1	1.93 (1.60–2.33)	1.26	1.21 (0.74–1.99)
≥ 20 cig./day	1.6	0.75 (0.36–1.53)	28.1	1.14 (0.93–1.40))	17.6	1.43 (1.12–1.81)	25.9	2.76 (2.24–3.41)	25.3	3.42 (2.58–4.53)	1.8	1.98 (1.01–3.89)
	**Neonatal death**		**Umbilical cord arterial pH < 7.1**		**Apgar at 5 min < 7**		**Neonatal infection**		**Hypoglycemia**		**Cerebral hemorrhage or convulsion**	
	**Prevalence %**	aOR (95% CI) ^a^	**Prevalence %**	aOR (95% CI) ^b^	**Prevalence %**	aOR (95% CI) ^b^	**Prevalence %**	aOR (95% CI) ^b^	**Prevalence %**	aOR (95% CI) ^b^	**Prevalence %**	aOR (95% CI) ^b^
Non-smokers	0.8	1	3.6	1	4.9	1	4.1	1	3.5	1	0.9	1
Smokers	0.9	0.89 (0.56–1.42)	2.3	0.65 (0.50–0.86)	5.3	0.97 (0.81–1.19)	4.5	1.04 (0.85–1.29)	4.4	1.18 (0.96–1.48)	0.8	0.75 (0.47–1.19)
< 10 cig./day	0.6	0.73 (0.34–1.60)	2.5	0.70 (0.48–1.02)	4.8	1.01 (0.76–1.34)	3.7	0.93 (0.68.1.28)	3.5	1.06 (0.76–1.49)	0.7	0.71 (0.35–1.47)
≥ 10 cig./day	1.1	0.99 (0.58–1.71)	2.2	0.62 (0.43–0.88)	5.7	0.95 (0.75–1.22)	5.1	1.13 (0.88–1.46)	5.0	1.27 (0.98–1.66)	0.9	0.77 (0.44–1.36)
≥ 20 cig./day	1.4	1.25 (0.56–2.79)	1.8	0.51 (0.26–0.99)	6.4	1.04 (0.71–1.54)	6.8	1.53 (1.05–2.22)	5.8	1.45 (0.96–2.19)	0.6	0.47 (0.15–1.50)
	**Jaundice**		**Neonatal anemia**		**Respiratory diseases **^**c**^		**Gastrointestinal diseases **^**d**^		**Transfers to NICU**		**Stay in NICU > 7 days**	
	**Prevalence %**	aOR (95% CI) ^b^	**Prevalence %**	aOR (95% CI) ^b^	**Prevalence %**	aOR (95% CI) ^b^	**Prevalence %**	aOR (95% CI) ^b^	Prevalence %	aOR (95% CI) ^b^	Prevalence %	aOR (95% CI) ^b^
Non-smokers	4.0	1	1.6	1	7.4	1	0.7	1	11.8	1	7.6	1
Smokers	4.5	1.09 (0.88–1.35)	2.0	1.12 (0.82–1.54)	10.1	1.32 (1.13–1.56)	1.3	1.63 (1.11–2.42)	16.5	1.44 (1.26–1.63)	11.9	1.64 (1.42–1.90)
< 10 cig./day	3.5	0.92 (0.66–1.29)	1.3	0.88 (0.51–1.50)	8.8	1.33 (1.05–1.69)	1.1	1.54 (0.86–2.79)	13.0	1.19 (0.98–1.45)	7.8	1.11 (0.88–1.42)
≥ 10 cig./day	5.4	1.21 (0.94–1.56)	2.5	1.28 (0.89–1.85)	11.1	1.32 (1.08–1.61)	1.5	1.69 (1.06–2.71)	19.3	1.63 (1.39–1.91)	15.2	2.07 (1.73–2.46)
≥ 20 cig./day	5.8	1.26 (0.83–1.90)	2.8	1.36 (0.76–2.44)	11.8	1.35 (0.98–1.88)	0.8	0.84 (0.30–2.35)	23.9	2.22 (1.74–2.83)	20.2	3.04 (2.34–3.96)

*IUGR* Intrauterine growth retardation, *NICU* Neonatal intensive care unit

^a^ Adjusted odds ratios for maternal age, origin, marital status, parity, previous pregnancy loss, education, professional status, psychosocial difficulties and health insurance, 95% confidence interval

^b^ Adjusted odds ratios for maternal age, origin, marital status, parity, previous pregnancy loss, education, professional status, psychosocial difficulties, health insurance and gestational age, 95% confidence interval

^c^ Respiratory distress syndrome, pulmonary infection, pneumothorax, apnea, hyaline membrane disease

^d^ Feeding difficulties, occlusive syndrome, digestive haemorrhage, necrotizing enterocolitis, diarrhea, vomiting

A significant dose–response relationship trend was identified between the number of daily cigarettes consumed and preterm birth (p_trend_ < 0.001), intrauterine growth restriction (p_trend_ < 0.001), birthweight < 2500 g (p_trend_ < 0.001), umbilical cord arterial pH below 7.1 (p_trend_ = 0.001), transfers to our neonatal intensive care unit (p_trend_ < 0.001), and neonatal intensive care unit admissions more than 7 days (p_trend_ < 0.001).

No trend was found for the other outcomes investigated: pre-eclampsia, increased rate of cesarean section, neonatal death, intrauterine death, APGAR score < 7 at 5 min, hypoglycemia, jaundice, neonatal anemia, neonatal infection, cerebral hemorrhage or convulsion, respiratory diseases, and gastrointestinal diseases.

## Discussion

As our database includes a sample of pregnant women from the 1997 to 2006, this likely explains why the rate of pregnant individuals who identify as smokers, 13.9%, is higher than the rate described in statistics from 2018, which are estimated to be 8.1% in Europe [5] and 7.2% in the USA [6].

Cigarette smoking has an impact on pregnancy with several adverse perinatal outcomes. In our study, cigarette use was strongly associated with preterm birth, lower birthweight, intrauterine growth restriction, transfers to the neonatal intensive care unit, and neonatal intensive care unit admissions > 7 days. All of the above associations have a dose–response relationship, with significant trend values. Our results align with those found in the literature [10–14, 16]. Intrauterine death was associated with heavy cigarette consumption (≥ 20/day), while other studies attributed intrauterine death with lower tobacco consumption [17–19]. Finally, smoking during pregnancy can induce neonatal pulmonary and gastrointestinal pathologies. Heavy cigarette consumption (≥ 20/day) also increases the risk of neonatal infections.

The mechanisms by which tobacco smoking result in adverse perinatal outcomes are complex. They may occur as a result of disruption of fundamental processes such as proliferation, apoptosis, and invasion of the trophoblasts during placental development. Alteration of the vascularization and the metabolism of the placenta may also be a cause [31].

The association between neonatal gastrointestinal pathology and smoking during pregnancy, as well as the association with neonatal infections, has been little studied until now. As a comparison, it has been shown that adult smokers are themselves more susceptible to bacterial or viral infections than non-smokers which may be due to alteration of the structural, functional, and immunological functions of the host defenses [32, 33].

Smoking during pregnancy may, however, also still be a protective factor. Cigarette use during pregnancy has been shown to reduce the risk of pre-eclampsia [21, 22] as was also identified in our study. The protective role of smoking can be partially explained by the effects of carbon monoxide, one of the products of tobacco combustion. Carbon monoxide inhibits the placental production of anti-angiogenic proteins such as sFlt1 or sEng, which play a role in the pathogenesis of preeclampsia. However, the pathogenesis of pre-eclampsia remains complex and is still not fully understood [34]. It may be worth mentioning that Luque-Fernandez et al. have partially explained the paradoxical phenomenon of this protective effect by studying prevalent cases at birth rather than all incident cases in a pregnancy cohort, which results in selection bias [35]. In our study, tobacco smoking was also a protective factor against the risk of umbilical cord arterial pH below 7.1. This phenomenon has been little studied. However, we will qualify our results by comparing them with those of Zaigham et al. whose prospective-observational cohort study of 308 patients showed no significant differences in pH values between smokers and non-smokers [36].

Our results do not suggest a significant association for some outcomes such as an APGAR score below 7 at 5 min, hypoglycemia, cerebral hemorrhage or convulsion, jaundice, and neonatal anemia.

With the proportion of pregnant smokers estimated to be 8.1% in Europe [5] and 7.2% in the USA [6] in 2018, it is clear that there is still much to be done in terms of prevention. Although low tobacco consumption is associated with less severe outcomes than heavy consumption, it is important to inform pregnant women that even at low doses, smoking has consequences for the fetus, in addition to the consequences on their own health. Effective interventions for smoking cessation during pregnancy include regular interval counseling and the provision of nicotine replacement therapy to patients who do not respond to counseling only [37]. The use of incentives to motivate smoking cessation also showed encouraging results [38].

The strength of our study is the analysis of multiple prospectively collected outcomes within a single, large cohort. It confirms the different outcomes studied separately in the literature but also demonstrated a dose–response effect, which has not been systematically evaluated [10–29].

Our research also contains some weaknesses. First, we did not assess a possible change in smoking during pregnancy and we also did not include the occasional smokers. This constitutes an information bias. By using a large available database, which was not designed specifically for this research, we were also unable to utilize a standardized questionnaire to assess cigarette consumption. Second, we did not assess passive smoking or secondhand exposure, which may also affect the fetus [39]. Furthermore, we did not take into account certain factors that could be confounding, such as alcohol or cannabis use [40, 41]. Information regarding other factors, such as comorbidities or concomitant medication use were not available and therefore were also not taken into account.

In addition, it is important to mention that some odds ratio confidence intervals are wide, especially for the subgroup of “ ≥ 20 cig/day”. This may be explained by the fact that this subgroup only includes 499 patients out of 19,554 patients. We thus acknowledge that some of the comparisons are underpowered, and therefore the lack of statistically significant relationships for some of the comparisons may not necessarily indicate that there is no relationship. Since the associations found in our study might be underestimated due to patients underreporting their consumption, this “ > 20 cig/day” group might represent the true impact of smoking during pregnancy. Indeed, about 24% of pregnant smokers stop smoking during pregnancy and up to 25% of pregnant smokers also misreport their actual tobacco consumption. This represents a possible classification bias.

We can also mention the lack of generalizability due to a localized sample. Finally, during the time period of our study (1997–2006), obstetrical management may have altered. This potential change was not taken into account as a covariate. Also, the rate of smoking in pregnancy has been declining [5]. Within the Swiss population, the latest existing data to our knowledge includes the years 2011–2016. The proportion of pregnant smokers during this time was estimated to be 6.8%, showing a decrease in consumption since the data collected for our research [42]. Although the estimate of association may hold, many characteristics of women in the study may not hold.

## Conclusion

Cigarette smoking during pregnancy is associated with several adverse perinatal outcomes. This relationship is often dose-dependent, as with preterm birth, birthweight < 2500 g, intrauterine growth restriction, transfers to neonatal intensive care unit, and neonatal intensive care unit admissions more than 7 days. Prevention among women must be further emphasized, as some adverse outcomes could be avoided by a smoke-free pregnancy.

## Data Availability

The datasets analysed during the current study are available from the corresponding author on reasonable request.
